# Comparative Analysis of SARIMA, Prophet, and a Diagnostic Decomposition–Correction Hybrid for Long-Horizon Lottery Sales Forecasting

**DOI:** 10.3390/e28030286

**Published:** 2026-03-03

**Authors:** Qian Cao, Zhenbang Sun, Huiyong Li

**Affiliations:** 1 School of Computer and Artificial Intelligence, Beijing Technology and Business University, Beijing 100048, China; 2331101004@st.btbu.edu.cn; 2 School of Artificial Intelligence (Institute of Artificial Intelligence), Beihang University, Beijing 100191, China; lihuiyong@buaa.edu.cn

**Keywords:** lottery sales forecasting, SARIMA, Prophet, diagnostic hybrid modeling, long-cycle seasonality

## Abstract

Accurate forecasting of lottery sales is crucial for strategic planning in volatile consumer markets driven by trend shifts, multi-scale seasonality, and calendar effects. This study proposes a Diagnostic Decomposition–Correction Hybrid (DDC-Hybrid) framework integrating Prophet and SARIMA through a residual diagnostics and correction pipeline. Specifically, Prophet is employed to model long-term trend changes and interpretable holiday impacts, while SARIMA is subsequently used to correct the residual series, capturing short-range temporal dependence that remains statistically significant after decomposition. From an information-theoretic perspective, the framework can be viewed as a two-stage uncertainty reduction process, where decomposition extracts low-frequency informative components and residual correction harvests remaining predictive information. Using monthly lottery sales in China (2008–2025), we conduct a comprehensive evaluation of SARIMA, Prophet, and the proposed hybrid approach. The DDC-Hybrid demonstrates improved predictive accuracy, yielding the lowest error rates. Beyond predictive accuracy, we further examine varying holiday effects through statistical testing. We also find that lottery sales contain a pronounced quadrennial (48-month) seasonal cycle associated with mega-sport events, which improves long-horizon stability. The results suggest that the proposed diagnostic hybrid modeling approach enhances forecasting accuracy and provides practical insights for lottery sales management.

## 1. Introduction

Lottery sales forecasting plays a critical role in government revenue management, operational budgeting, and demand planning in the gambling industry. As a popular form of entertainment, lotteries attract a broad consumer base and serve as a significant source of public revenue. According to the Ministry of Finance of the People’s Republic of China, total lottery sales in China from January to August 2024 reached 417.5 billion CNY, marking an 11.1% year-on-year increase [1]. Among these, sports lottery sales accounted for 277.1 billion CNY, and welfare lottery sales amounted to 140.3 billion CNY. This massive market scale highlights the economic and social significance of lotteries. However, forecasting lottery sales is fundamentally challenging due to the coexistence of structural trend shifts, multi-scale seasonality, and event-driven demand shocks, which jointly induce non-stationary and partially explainable dynamics.

Despite the massive economic scale of the lottery market, academic research specifically targeting lottery sales forecasting remains disproportionately scarce compared to other financial or retail sectors. The majority of existing studies predominantly rely on traditional econometric frameworks or singular statistical models. While these conventional methods establish a baseline, they often yield suboptimal performance when capturing the unique complexities of the lottery market—specifically the interplay of long-cycle events and abrupt policy-driven shocks. Among these traditional approaches, the Seasonal Autoregressive Integrated Moving Average (SARIMA) model is one of the most widely applied methods. However, it often struggles with complex nonlinear fluctuations or sudden external shocks. In contrast, Prophet, developed by Facebook in 2017, was designed to handle multiple seasonalities, holiday effects, and missing data, making it a flexible forecasting tool. Despite these advantages, both SARIMA and Prophet exhibit complementary weaknesses: SARIMA struggles under structural changes and nonlinear fluctuations, while Prophet often leaves statistically meaningful short-term dependence in the residuals, leading to long-horizon forecast errors.

To address these limitations, we propose a Diagnostic Decomposition–Correction Hybrid framework (DDC-Hybrid), which reframes hybrid modeling not as a simple ensemble, but as a residual diagnostics and correction pipeline. Specifically, Prophet is first employed to learn interpretable global structures (trend, seasonality, and holiday impact). Residual diagnostics (ACF/PACF and Ljung–Box test) are then used to verify the existence of temporal dependency that remains unexplained. Subsequently, SARIMA is applied to the residual series to model and forecast short-term dependence, and the predicted residuals are added back to Prophet forecasts to obtain the final prediction. This design enhances both interpretability and long-horizon stability in volatile lottery sales forecasting.

From an information-theoretic standpoint, lottery sales dynamics can be characterized by time-varying uncertainty under trend shifts, multi-scale seasonality, and calendar-driven demand shocks. In this context, entropy provides a natural language to describe the uncertainty level of the sales-generating process, where higher entropy corresponds to less predictable demand dynamics. In such scenarios, forecasting is not only a point-estimation task, but also a process of extracting predictive information from historical observations to reduce uncertainty about future sales. Prophet can be interpreted as extracting low-frequency informative components (trend, multiple seasonalities, and holiday effects), while the residual series represents the unexplained part of the signal. If the residuals are not white noise and still exhibit statistically significant dependence, this indicates that the model has not fully removed predictable information. Therefore, the proposed DDC-Hybrid framework can be regarded as a two-stage information extraction pipeline, where SARIMA is employed to capture remaining short-range predictive information embedded in residual dependence, thereby further reducing forecast uncertainty and improving long-horizon stability.

The contributions of this paper are summarized as follows: (1) We propose a structured forecasting workflow by providing explicit diagnostic justification for the model integration. Our pipeline rigorously validates the necessity of residual correction through statistical hypothesis testing, ensuring a mathematically grounded combination of Prophet and SARIMA. (2) We identify and validate a pronounced quadrennial (48-month) event-induced seasonality in Chinese lottery sales and benchmark seasonal cycle settings to demonstrate its practical value for long-horizon forecasting. (3) We provide an interpretable and statistically grounded quantification of public holiday effects, highlighting heterogeneous impacts that can support policy-makers and lottery administrators in demand planning and resource allocation.

The remainder of the paper is organized as follows. Section 2 reviews related time series forecasting models, including classical statistical approaches, machine learning methods, and hybrid strategies. Section 3 presents the dataset, preprocessing procedures, and implementation details of SARIMA, Prophet, and the DDC-Hybrid. The analysis then proceeds in Section 4, which presents the experimental results and offers a comparative discussion of model performance, including an evaluation of holiday and seasonal effects. Finally, Section 5 concludes the paper with a summary of findings, model limitations, and directions for future research.

## 2. Literature Review

Existing studies have highlighted the evolution of forecasting techniques from classical statistical approaches to modern machine learning and hybrid models. The following subsections provide an overview of these methodologies and their relevance to lottery sales forecasting.

### 2.1. Classical and Machine Learning Models for Time Series Forecasting

Time series forecasting has long been a core method in data-driven decision-making, particularly in sectors such as finance, energy, health, and commerce. Among classical methods, the ARIMA and SARIMA models remain foundational for capturing trends and seasonal patterns in univariate time series data [2,3]. These models have been successfully applied to tasks such as electricity load prediction [4], stock market volatility [5], and retail forecasting [6].

Machine learning methods, particularly Long Short-Term Memory (LSTM) networks, have emerged as promising alternatives. LSTM models are capable of learning nonlinear dependencies and long-range temporal structures, achieving notable performance in applications such as cryptocurrency forecasting [7], ocean weather modeling [8], and short-term financial predictions [9]. Despite their effectiveness, these models often require extensive parameter tuning and significant computational resources.

The Prophet model, developed by Facebook, offers a practitioner-friendly solution by combining trend, seasonality, and holiday effects into a decomposable framework [10]. It is widely adopted in real-world business cases like website traffic prediction [11], supermarket sales forecasting [12], and financial time series forecasting [13].

### 2.2. Hybrid Forecasting Models

Increasingly, researchers advocate hybrid forecasting models that combine statistical precision with machine learning flexibility. One popular structure is the SARIMA-Prophet hybrid, which has demonstrated high accuracy and stability in contexts such as epidemic trend forecasting [14] and e-commerce product sales [15]. Other hybrid models integrate neural networks with SARIMAX, showing superior adaptability in handling multiple seasonalities and irregular patterns [16].

Advanced ensemble strategies, including L1-norm-based combinations, have been shown to enhance robustness against outliers and improve generalization [17]. Meanwhile, the NeuralProphet model merges the structure of Prophet with neural components to increase interpretability while maintaining performance on large-scale datasets [18].

Empirical evaluations have confirmed the benefits of hybrid approaches. Comparative studies involving ARIMA, LSTM, and Prophet models in contexts such as monkeypox outbreak prediction [19] and COVID-19 trend analysis [20] consistently show that hybrid or ensemble approaches yield more reliable results across forecasting horizons.

### 2.3. Applications to Lottery Sales Forecasting

In the specific context of lottery sales forecasting, several studies highlight the importance of incorporating socio-economic, regional, and behavioral factors. Jia and Xie [21] used residual principal component analysis to explore the economic indicators influencing provincial lottery sales in China. Wei and Lei [22] employed spatial regression to reveal regional disparities, while Han [23] and Liu [24] modeled factors influencing lottery ticket purchases and bonus distribution using panel and econometric methods.

Earlier foundational works by Hu and Qian [25], Zhang and Zeng [26], and Shen and Zhou [27] proposed mathematical frameworks for understanding lottery purchase strategies, optimizing incentives, and simulating ticket sales behavior.

Together, these studies emphasized that accurate forecasting in behavioral and consumer-driven domains like lotteries benefits significantly from hybrid modeling—leveraging statistical insights, machine learning adaptability, and contextual knowledge to drive predictive accuracy and actionable outcomes. To contextualize our contribution, Table 1 provides a comparative summary of the key forecasting models discussed in the literature and contrasts them with the proposed DDC-Hybrid framework.

## 3. Methodology

This study followed an evaluation protocol, including data collection, preprocessing, model implementation, and performance evaluation. The dataset was first prepared to ensure accuracy, followed by the application of SARIMA, Prophet, and DDC-Hybrid. Finally, their forecasting performance was assessed using key evaluation metrics.

### 3.1. Data Collection and Preprocessing

The dataset used in this study consists of historical lottery sales data for China, covering the period from July 2008 to December 2025 [28]. The dataset is chronologically partitioned into a training set and a testing set. The training set comprises data from July 2008 to August 2024, which is utilized for model fitting and parameter estimation. The subsequent period, from September 2024 to December 2025, serves as the out-of-sample testing set.

To ensure data consistency, preprocessing was conducted, particularly addressing extreme outliers caused by the COVID-19 pandemic in February and March 2020. To prevent these outliers from distorting the forecasting models, linear interpolation was selected. The rationale for this approach is twofold. First, the precipitous drop in sales was primarily driven by an administrative shutdown rather than a shift in consumer preference. Due to strict lockdown measures, physical lottery terminals were closed, rendering the data points as indicators of ‘system unavailability’ rather than true market demand. Second, our objective is to model long-term cyclical dynamics. Retaining these extreme outliers would act as high-leverage points, severely distorting the estimation of regular seasonal parameters. By interpolating, we construct a ‘counterfactual baseline’ representing the market’s intrinsic trajectory absent the physical blockade, thereby ensuring robust parameter estimation for long-horizon forecasting under normal operational conditions.

**Figure 1:**
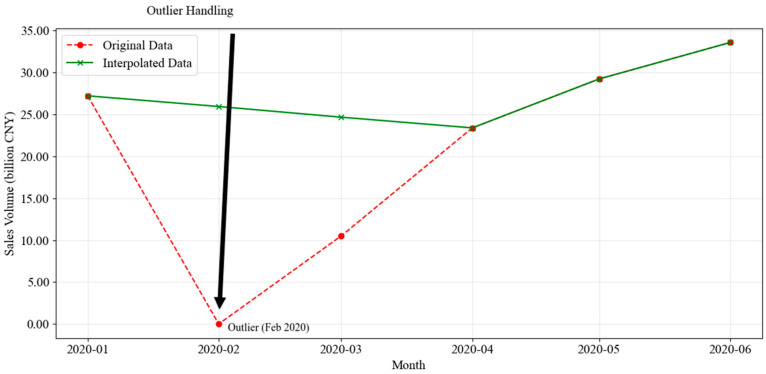
Outlier Handling Comparison (February–March 2020).

Figure 1 illustrates the comparison between the original data and the interpolated data for February and March 2020. The red dashed line highlights the extreme drop in sales, while the green line represents the corrected values after interpolation. The black arrow marks the most significant outlier in February 2020. This adjustment ensured the dataset remains smooth and reliable for forecasting purposes.

In addition to outlier handling, the dataset underwent a stationarity test to determine its suitability for time series modeling. Many forecasting models rely on stationary data. For models like SARIMA, it is presumed that non-stationarity can be resolved through differencing operations. The Augmented Dickey–Fuller (ADF) test [29] was performed on the dataset, and the results indicated that the original sales data had a *p*-value of 0.8061, confirming that it was non-stationary. To address this, a logarithmic transformation was applied to stabilize variance, which reduced the *p*-value to 0.3517 but did not fully eliminate the trend. Subsequently, first-order differencing was performed, which lowered the *p*-value to 0.0246, confirming that the transformed dataset was stationary.

**Figure 2:**
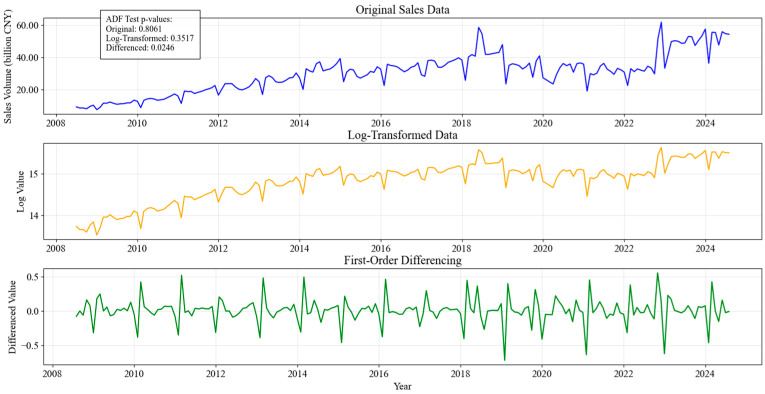
ADF Test and Data Transformation Effects.

Figure 2 visualizes the impact of these transformations. The top panel represents the original sales data, displaying a strong upward trend, indicating non-stationarity. The middle panel presents the log-transformed data, which helps stabilize variance but does not entirely remove the trend. The bottom panel illustrates the first-order differenced data, which effectively removes the trend and achieves stationarity. These preprocessing steps were crucial in preparing the dataset for modeling, ensuring that the forecasting algorithms could accurately capture underlying seasonal and cyclical patterns.

By addressing outliers and ensuring stationarity, the dataset was refined to provide robust and meaningful forecasting results. These transformations helped minimize bias in the model’s predictions, which improves data consistency and supports stable long-horizon forecasting.

### 3.2. Model Selection and Implementation

This study evaluated three forecasting models: SARIMA, Prophet, and DDC-Hybrid combining the strengths of both SARIMA and Prophet. Each model was chosen for its distinctive advantages in time series forecasting.

#### 3.2.1. SARIMA Model

The Seasonal Autoregressive Integrated Moving Average (SARIMA) model is widely used for time series forecasting, particularly when data exhibits both trend and seasonality [3]. As verified in Section 3.1, the original sales data exhibited non-stationarity. Therefore, we applied logarithmic transformation to stabilize variance. Subsequently, first-order differencing was employed to eliminate the trend and achieve the stationarity required for the autoregressive and moving average components.

To determine the optimal model structure, we employed the auto_arima function [30]. We utilized the Corrected Akaike Information Criterion (AICc) for model selection to mitigate the risk of overfitting. The search space for non-seasonal parameters (p, q) was constrained to [0, 5], with a maximum total order of 5 to prevent overfitting. Table 2 presents the top 10 SARIMA models ranked by AICc value.

From the models evaluated, the SARIMA(0,1,1)(2,0,0)[48] model with intercept achieved the lowest AICc value of −187.478, where 48 denotes the seasonal period. Beyond minimizing the information criterion, this selection strictly adheres to the principle of parsimony. This model was therefore selected for forecasting.

**Figure 3:**
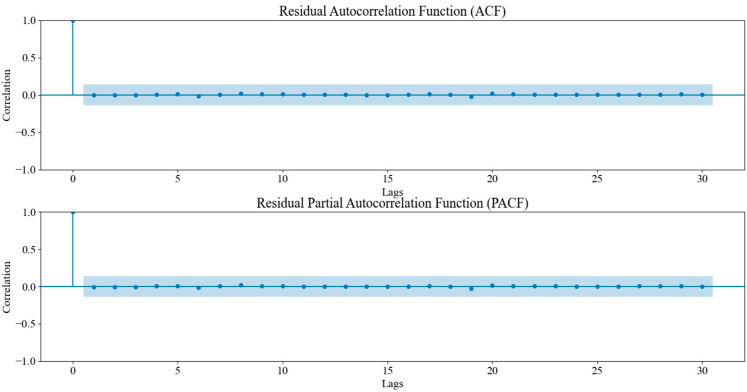
Residual ACF and PACF of the SARIMA model.

The SARIMA model was trained on the transformed dataset with a seasonal period of 48 months to capture the quadrennial FIFA World Cup cycle, a configuration supported by seasonality sensitivity analysis. Model selection relied on maximizing R^2^ and minimizing MAPE, confirming that the 4-year cycle best accounted for periodic sales surges. Subsequent residual analysis (Figure 3) showed no significant autocorrelation in the ACF and PACF plots, validating the model’s efficacy in handling linear seasonal patterns. However, SARIMA exhibited limitations in capturing complex nonlinear variations caused by external shocks or policy shifts. In the ACF/PACF plots, the points/vertical bars represent the estimated autocorrelation (ACF) and partial autocorrelation (PACF) coefficients at each lag, while the blue shaded band indicates the 95% confidence bounds (approximately ±1.96/√N). Coefficients falling outside the band suggest statistically significant residual correlation.

#### 3.2.2. Prophet Model

The Prophet model exhibited remarkable suitability for time series data characterized by pronounced seasonal patterns and holiday effects, rendering it an exemplary choice for forecasting lottery sales [10]. To improve forecasting accuracy, Chinese public holidays were explicitly integrated as regressor components. Each holiday was assigned specific dates and impact windows, as detailed in Table 3.

Prophet incorporated these holidays into the forecasting framework by modeling their effects as additional components. The total sales $y\left(t\right)$ at time t in Prophet can be expressed as the sum of several components:

(1) $$y\left(t\right)=g\left(t\right)+s\left(t\right)+h\left(t\right)+e$$

The trend component $g\left(t\right)$ models the overall growth or decline in sales over time and is described by a piecewise linear (or logistic) growth function,

(2) $$g\left(t\right)=\frac{C}{1+\exp\left(-k\left(t-t_{0}\right)\right)}$$

where C represents the carrying capacity (the maximum possible value of the trend), k is the growth rate, $t_{0}$ is the inflection point, and t is the time.

The seasonal component $s\left(t\right)$ captures periodic fluctuations in sales (e.g., yearly cycles). Prophet models seasonality using a Fourier series to represent these cycles,

(3) $$s\left(t\right)=\sum_{k=1}^{K}\left(\alpha_{k}\cos\left(\frac{2\pi kt}{P}\right)+\beta_{k}\sin\left(\frac{2\pi kt}{P}\right)\right)$$

where P denotes the seasonal period and K is the number of Fourier terms used. In this study, an additional long-cycle seasonality term with a 48-month period was introduced to account for quadrennial event-driven fluctuations.

The holiday effects h(t) account for the influence of specific public holidays on sales. Each holiday’s effect is modeled with an indicator function $I(t,{Holiday}_{i})$, which takes the value 1 if t falls within the holiday window, and 0 otherwise. $\gamma_{i}$ represents the impact of holiday i on the sales, quantifying the average deviation during the holiday period. The holiday effect is then expressed as:

(4) $$h\left(t\right)=\sum_{i=1}^{n}I\left(t,{Holiday}_{i}\right)\cdot \gamma_{i}$$

Hyperparameters were optimized via grid search to balance flexibility and generalization. The search space for changepoint_prior_scale was restricted to [0.03, 0.05, 0.08], while K was explored within [5, 10, 15]. The grid search results are summarized in Table 4, which shows that the lowest MAPE was achieved when changepoint_prior_scale = 0.05 and K = 15.

When the holidays and seasonal components are included, Prophet uses the historical sales data to train the model, learning the trends, seasonal effects, and holiday adjustments. The model was then used to generate forecasts for the next 48 months.

#### 3.2.3. DDC-Hybrid

The Diagnostic Decomposition–Correction Hybrid (DDC-Hybrid) was designed to integrate the strengths of both individual models, combining the ability of Prophet to capture trends and seasonality with the strength of SARIMA in modeling residual structures. This integration facilitated enhancing forecasting accuracy by addressing the limitations of each model separately, thereby amplifying their combined efficacy.

The first step in building the DDC-Hybrid was fitting the Prophet model to the lottery sales data. As in the standalone Prophet model, Chinese public holidays were incorporated into the model to enhance its ability to capture fluctuations in sales caused by these events. Additionally, a 4-year seasonal cycle was introduced using a Fourier term to account for the impact of the World Cup, which had a periodic effect on lottery sales every four years. The primary role of the Prophet model in this diagnostic pipeline was to fit the overall trend and major seasonal variations, generating an initial set of predictions.

Before applying the SARIMA model to the residuals, we conducted a diagnostic check on the residuals from the Prophet model. These checks were essential to determine whether the residuals exhibited any significant autocorrelation.

**Figure 4:**
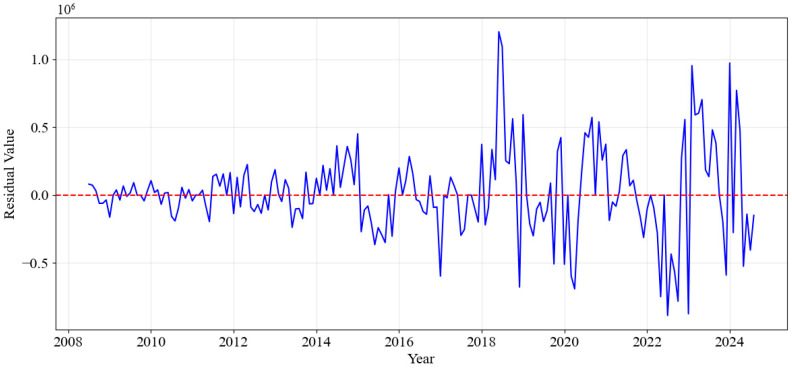
Residuals Over Time.

We analyzed the time series of the residuals from the Prophet model and observed that the residuals exhibited significant fluctuations over time, especially after 2018. In this period, both the amplitude and frequency of the fluctuations increased, suggesting that the model’s prediction errors were less stable during these times. This indicated the possibility of underlying temporal patterns that the Prophet model did not capture effectively, leading to increased forecasting errors in these periods. Figure 4 shows the residuals over time, highlighting the fluctuation patterns.

**Figure 5:**
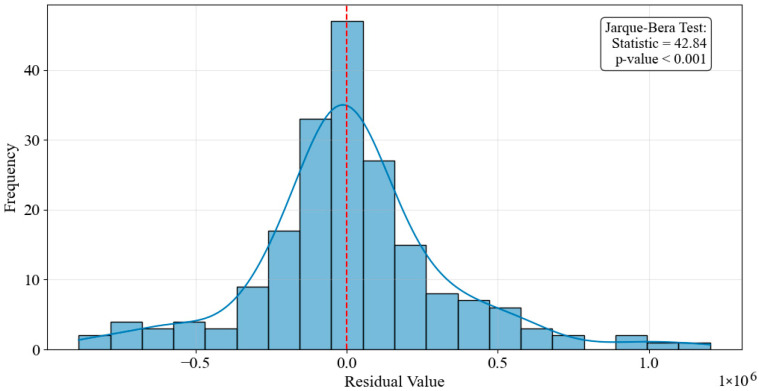
Distribution of Residuals.

We also examined the distribution of the residuals. The analysis revealed that while the residuals were generally centered around zero, they exhibited a concentration near zero with a long-tailed distribution on both sides, particularly on the right side (the direction of high residual values). This indicated the presence of some extreme values, suggesting that the residuals did not follow a perfect normal distribution. To formally assess this, the Jarque–Bera test was performed, yielding a test statistic of 42.84 with a *p*-value < 0.001, which rejects the null hypothesis of normality. This quantitative evidence confirms a significant deviation from the normal distribution, primarily driven by the observed skewness and kurtosis. This might have implied that the model had not fully accounted for extreme values, or that there are underlying anomalous fluctuations in the data that had not been well captured by the Prophet model. Figure 5 presents the distribution of the residuals, showing the skewness and the long-tail nature.

**Figure 6:**
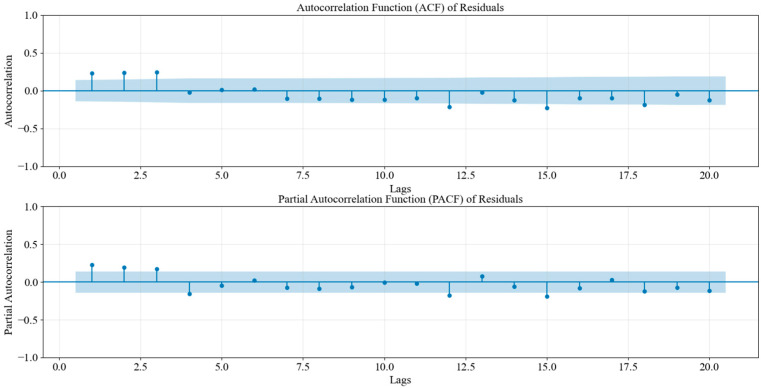
Prophet residual ACF/PACF (see Section 3.2.1 for definitions).

To investigate the autocorrelation in the residuals, we plotted the ACF and PACF. These plots showed that the autocorrelation coefficients for lower-order lags (such as lags 1 to 3) exceeded the confidence intervals, which indicated significant autocorrelation at these lags. This suggested that the residuals were not fully random and might have still contained some underlying patterns. This implied that the model had not fully captured all the dynamics in the original data, and there might have been valuable information that had not been fully extracted. Figure 6 shows the ACF and PACF of the residuals, illustrating the significant autocorrelation at several lags.

The Ljung–Box Q test was performed to test the null hypothesis that the underlying error series is white noise, based on the observed residuals. At lag 1, the Ljung–Box statistic was 10.2493 with a *p*-value of 0.0014, well below 0.05. From lags 2 through 20, the *p*-values were consistently below 0.001, which indicated statistically significant autocorrelation across these lags. These results rejected the white-noise hypothesis for the residual series and showed that meaningful temporal structure remained after the Prophet fit, which justified modeling the residuals with SARIMA.

To provide additional intuition for this diagnostic result, we interpret the residual dependence from an information-theoretic perspective. In forecasting, structural decomposition can be viewed as extracting informative components that reduce entropy (uncertainty) about future observations. Therefore, if the Prophet residuals exhibit statistically significant autocorrelation rather than white-noise behavior, this implies that the residuals still carry exploitable predictive information not captured by the decomposable trend–seasonality–holiday structure. This motivates a second-stage correction model to further capture the remaining temporal dependence and improve long-horizon stability.

**Figure 7:**
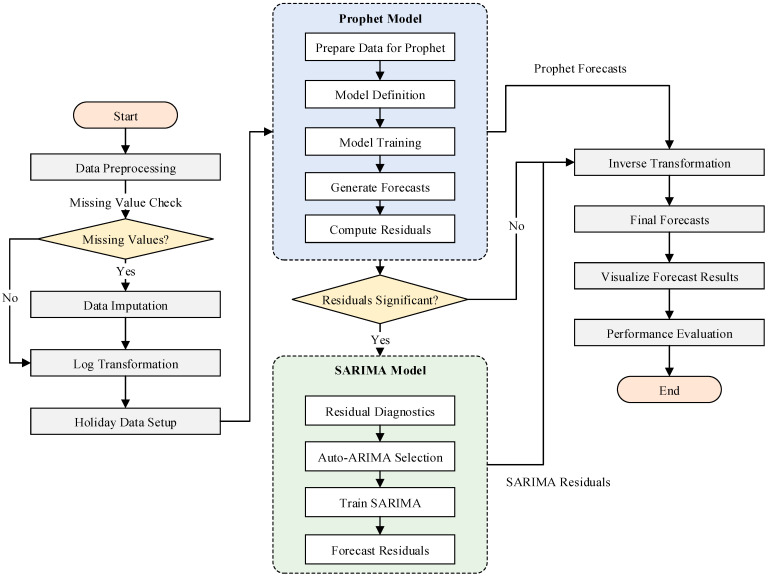
Process Flow of DDC-Hybrid.

Next, a diagnostic residual-correction stage was performed by applying SARIMA to the residuals produced by the Prophet decomposition. The residuals were computed as the difference between the actual sales data and the values predicted by Prophet. These residuals represented the patterns that Prophet was unable to capture effectively. The SARIMA model was then trained on these residuals to model any remaining structures, such as short-term dependencies or cyclical variations that were not accounted for by Prophet. Once fitted, SARIMA was used to forecast future residuals for the prediction period. The process flow of the DDC-Hybrid forecasting pipeline is summarized in Figure 7.

The proposed residual diagnostics and correction pipeline generated the final forecasts by adding the SARIMA-predicted residuals back to the base Prophet forecasts. Specifically, the residuals predicted by SARIMA were added back to the base Prophet forecasts, yielding the final adjusted sales predictions. The inverse-log transformation was subsequently applied to revert the values to their original sales scale. This step ensured that the final forecasted sales figures were interpretable and directly comparable to the observed historical data.

The DDC-Hybrid integrates Prophet’s interpretable structural modeling (trend/seasonality/holiday effects) with SARIMA-based residual correction, yielding a structure-aware and statistically justified hybrid forecasting framework. However, the effectiveness of this combination depended on how well SARIMA captured the residual patterns left unaccounted for by Prophet.

Finally, to address the heteroskedasticity observed in the residuals, specifically the increased variance in the post-2018 period, we introduced a GARCH(1,1) module as the final stage of the pipeline. While SARIMA captures the conditional mean, GARCH models the conditional variance of the final residuals. This allows for the construction of dynamic 95% confidence intervals that automatically widen during volatile periods and narrow during stable periods, providing a more rigorous risk assessment than static error bounds.

### 3.3. Evaluation Metrics

To evaluate the overall performance of the SARIMA, Prophet, and DDC-Hybrid, four key metrics were chosen: R-squared (R^2^), Mean Absolute Percentage Error (MAPE), Symmetric Mean Absolute Percentage Error (SMAPE) and Mean Squared Error (MSE). These metrics assessed different aspects of forecast accuracy, including model fit and forecasting error.

R-squared (R^2^) measures the proportion of variance in the observed data explained by the model. Higher R^2^ values indicate stronger explanatory power on the training data. R^2^ is expressed as:

(5) $$R^{2}=1-\frac{\sum_{t=1}^{n}{\left(Y_{t}-{\hat{Y}}_{t}\right)}^{2}}{\sum_{t=1}^{n}{\left(Y_{t}-\bar{Y}\right)}^{2}}$$

MAPE (Mean Absolute Percentage Error) calculates the average of the absolute percentage differences between the actual and predicted values. It is widely used because it expresses errors in percentage terms. The formula for MAPE is:

(6) $$MAPE=\frac{1}{n}\sum_{t=1}^{n}\left|\frac{Y_{t}-{\hat{Y}}_{t}}{Y_{t}}\right|\times 100\%$$

SMAPE (Symmetric Mean Absolute Percentage Error) is an alternative to MAPE that treats over- and under-forecasting equally. SMAPE is defined as:

(7) $$SMAPE=\frac{1}{n}\sum_{t=1}^{n}\frac{\left|Y_{t}-{\hat{Y}}_{t}\right|}{\frac{\left|Y_{t}\right|+\left|{\hat{Y}}_{t}\right|}{2}}\times 100\%$$

MSE (Mean Squared Error) is introduced as a metric for absolute error variance to further evaluate the robustness of the models and penalize larger forecasting errors. The MSE is defined as:

(8) $$MSE=\frac{1}{n}\sum_{t=1}^{n}{\left(Y_{t}-{\hat{Y}}_{t}\right)}^{2}$$

These four metrics collectively provide a comprehensive evaluation of model performance. R^2^ evaluates the model’s goodness of fit, while MAPE and SMAPE focus on forecasting accuracy, with SMAPE offering a more robust measure when actual values are close to zero. The inclusion of MSE provides a standard measure of absolute error variance, effectively penalizing large-scale deviations and ensuring the stability of the long-horizon forecast.

To quantify the reduction in aleatoric uncertainty at each modeling stage, we compute the differential entropy of the residual series. Assuming the residuals follow a Gaussian distribution with variance $\sigma^{2}$, the entropy is estimated as:

(9) $$H\left(X\right)\;\approx \;\frac{1}{2}\;ln(2\pi e\sigma^{2})$$

We acknowledge that while the residual series may exhibit deviations from strict normality, this closed-form expression serves as a computationally efficient proxy for evaluating the relative reduction in uncertainty and dispersion across different modeling stages.

Building on the methodology described above, the following section presents the experimental results and performance comparisons of the SARIMA, Prophet, and DDC-Hybrid. These results are discussed in the context of their applicability to long-term lottery sales forecasting.

## 4. Results and Discussion

To verify the effectiveness of the proposed hybrid forecasting approach, we conducted a series of experiments comparing the performance of SARIMA, Prophet, and their hybrid combination. The models were evaluated using key performance metrics to determine their effectiveness in predicting lottery sales. The following subsections provide a detailed analysis of each model’s accuracy and limitations.

### 4.1. Model Performance Comparison

The performance of SARIMA, Prophet, and the DDC-Hybrid was evaluated using R^2^ for in-sample fit, and MAPE, SMAPE, and MSE for out-of-sample forecasting accuracy. These evaluation metrics provided a comprehensive understanding of each model’s ability to fit the historical data and predict future sales. Table 5 presents the evaluation results for each model, highlighting the differences in forecasting accuracy.

First, in terms of historical data fitting, all three models exhibited explanatory power, indicated by high R^2^ values. This confirms that the models captured the underlying growth trends and seasonal patterns of the Chinese lottery market over the past 15 years.

The primary evaluation focuses on the out-of-sample testing period, which is critical for verifying the model’s generalization capability under the recent market regime shift. As shown in Table 5, the DDC-Hybrid model achieved the lowest error metrics, recording the lowest MAPE (6.51%), SMAPE (6.23%) and MSE(18.23). To further assess robustness under dynamic updating conditions, we conducted an Expanding Window (rolling origin) analysis. In this blind testing scenario, the standard Prophet model suffered from significant lag effects, yielding a high MAPE of 14.87%, as it struggled to adapt to the structural break without expert intervention. In contrast, the DDC-Hybrid model reduced this error to 10.88%. This comparison highlights that even in a rolling forecast setting, the DDC framework’s correction mechanism offers better stability and adaptability than the standalone trend model.

To assess the statistical significance of the forecast improvements, we conducted the Diebold-Mariano (DM) test. The test was performed on the multi-step forecast horizon (h=1 to 16) covering the out-of-sample period. The results yielded a DM statistic of 0.5136 and a *p*-value of 0.6075. While the *p*-value exceeds the 0.05 threshold, indicating that the improvement is not statistically significant at the 5% level, this outcome is likely attributable to the limited power of the test given the relatively short evaluation period (16 observations). Nonetheless, the consistent reduction in error metrics across the test set suggests practical utility and improved robustness in capturing the complex market dynamics.

To further quantify the model’s capacity for uncertainty reduction from an information-theoretic perspective, we calculated the differential entropy of the residual series at each stage of the DDC framework. The analysis reveals that the entropy of the original logarithmic sales series was 0.6949 nats. In the first stage, after removing trend and seasonal components via Prophet, the residual entropy dropped significantly to −0.9123 nats, representing a substantial reduction of 1.6072 nats. This suggests that Prophet successfully captured the majority of deterministic patterns. In the second stage, the subsequent SARIMA correction further reduced the entropy to −1.0736 nats. The cumulative entropy reduction of 1.7685 nats throughout the modeling pipeline provides comparative quantitative evidence supporting the efficacy of the DDC-Hybrid framework as a stepwise process for extracting information and minimizing aleatoric uncertainty.

#### 4.1.1. SARIMA Model Performance

**Figure 8:**
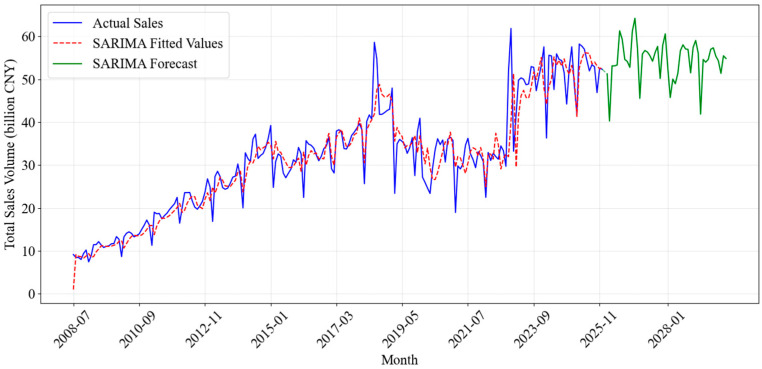
Fitting and Forecasting Results of the SARIMA Model.

SARIMA demonstrated moderate effectiveness in capturing baseline seasonal patterns typical of lottery sales. However, its predictive accuracy significantly diminished over longer horizons, as the linear framework failed to adapt to complex non-linear fluctuations and sudden structural shifts, resulting in increasing divergence from actual sales trajectories. The forecast performance of the SARIMA model is visualized in Figure 8.

#### 4.1.2. Prophet Model Performance

**Figure 9:**
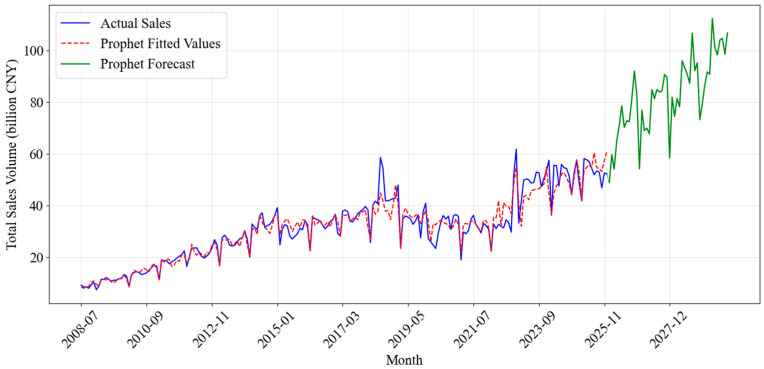
Fitting and Forecasting Results of the Prophet Model.

Prophet demonstrated a significant advancement over SARIMA by effectively modeling non-linear trends and complex seasonalities. Its flexible component-based structure allowed for the explicit integration of holiday effects—a capability SARIMA lacks—resulting in a closer alignment with actual sales spikes. As demonstrated in Figure 9, the Prophet model’s forecast aligns more closely with the actual sales data, especially during periods of high variation. This represented a marked improvement over the SARIMA model, which struggled to capture such fluctuations accurately.

Despite these advantages, the Prophet model undeniably possessed its limitations. While it exhibited commendable performance in capturing seasonal trends and long-term forecasts, it still struggled with extreme short-term fluctuations and highly irregular sales events. These instances, which might have been due to abrupt market changes or one-off events, were less accurately modeled by Prophet, as the model relied on historical data patterns for predictions.

#### 4.1.3. DDC-Hybrid Performance

The DDC-Hybrid demonstrated the lowest forecast errors among the three models evaluated, leveraging the strengths of both SARIMA and Prophet to achieve more accurate forecasts. By combining Prophet’s ability to capture long-term trends, seasonal effects, and holiday effects with SARIMA’s capacity to refine residuals, the DDC-Hybrid produced improved accuracy.

The model’s high in-sample R^2^ (0.9515) confirms its strong capability to explain historical variations. Meanwhile, the lowest out-of-sample MAPE (6.51%) and SMAPE (6.23%) demonstrate its forecasting accuracy on unseen data. These indicators illustrated the DDC-Hybrid’s ability to not only fit the historical data more closely but also to generate more reliable and accurate predictions for future sales. The high R^2^ value indicated that a larger proportion of the variance in the sales data was explained by the DDC-Hybrid, which reflected its ability to capture the underlying data structure.

**Figure 10:**
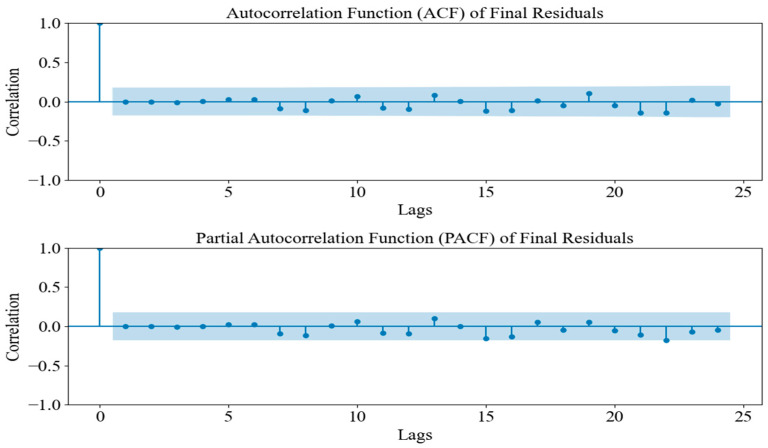
Residual ACF/PACF after SARIMA correction (see Section 3.2.1 for definitions).

To further validate the effectiveness of the DDC-Hybrid, residual analysis was conducted. The ACF and PACF plots were generated to examine whether the residuals exhibited no statistically significant autocorrelation. If the residuals exhibited no significant autocorrelations beyond the confidence interval, this indicated that SARIMA successfully captured all remaining structures, ensuring that no systematic patterns remained unmodeled. Figure 10 presents the ACF/PACF analysis of the DDC-Hybrid residuals after SARIMA correction.

The results of the ACF and PACF plots indicated that all lags fell within the confidence bounds, suggesting that no statistically significant autocorrelation remained in the corrected residuals. Consequently, this validation further strengthened the argument that the DDC-Hybrid provided the most reliable and accurate long-term forecasts.

**Figure 11:**
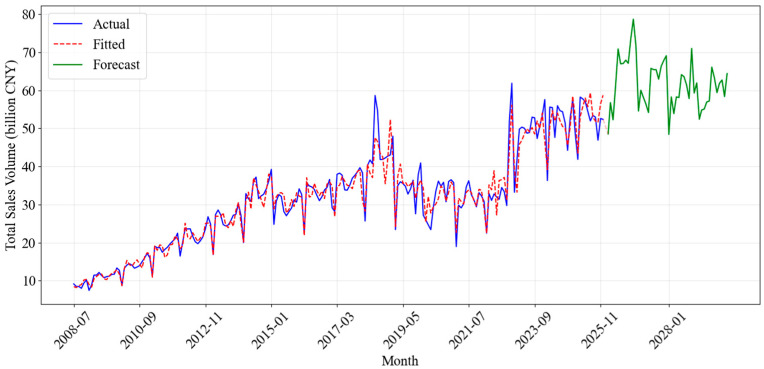
Fitting and Forecasting Results of the DDC-Hybrid.

This improved performance suggests that the residual correction mechanism successfully captured the nuance short-term dependencies and cyclical deviations that the standalone Prophet model overlooked, thereby enhancing overall predictive precision. Figure 11 shows the final forecast results generated by the DDC-Hybrid.

To ensure a rigorous assessment of forecast uncertainty, we further examined the higher-order properties of the residuals. We performed an ARCH test on the final SARIMA residuals, which rejected the null hypothesis of homoskedasticity (*p* < 0.05), confirming the presence of volatility clustering. The subsequent GARCH(1,1) estimation yielded a highly significant persistence parameter (β = 0.9483, *p* < 0.001) and a sum of coefficients (α + β = 0.985) close to unity. This high persistence empirically validates that the variance of lottery sales residuals is not constant but exhibits time-varying memory, justifying the need for dynamic volatility scaling.

**Figure 12:**
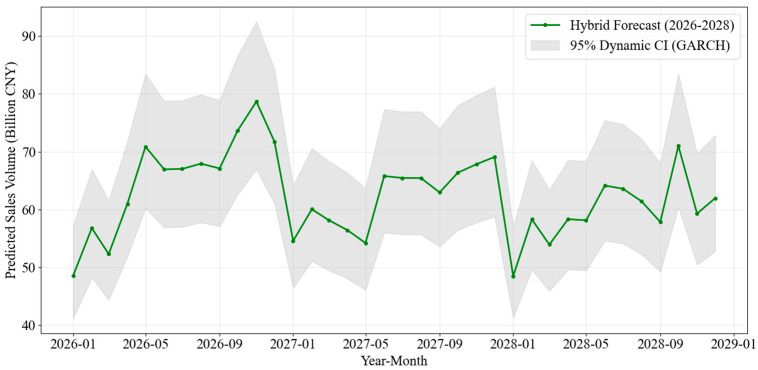
Forecasted Lottery Sales with 95% Dynamic Confidence Interval (2026–2028).

Following the model validation, we extended the forecast horizon to the 2026–2028 period. Figure 12 provides a focused, high-resolution visualization of this future trajectory, explicitly incorporating dynamic 95% confidence intervals derived from the GARCH volatility forecasts. It visualizes the expanding uncertainty bands during mid-2026 and 2027. The forecast projects total sales to reach approximately 782.35 billion CNY by 2026, with the dynamic intervals offering a precise quantification of the expanding downside and upside risks.

### 4.2. Discussion

#### 4.2.1. Influence of Holidays

**Figure 13:**
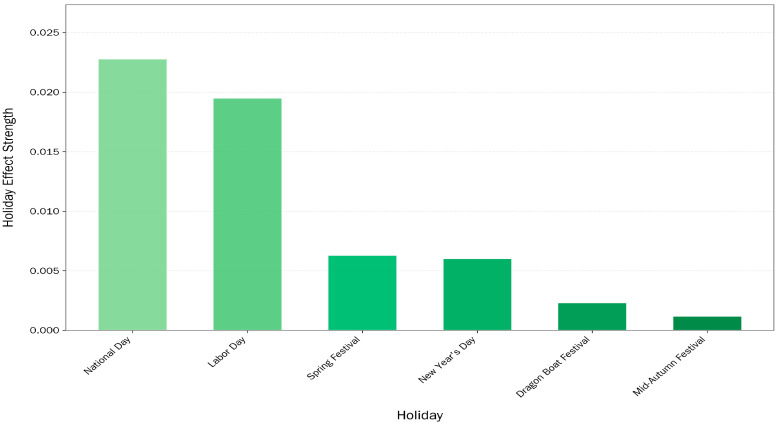
Average Holiday Effects on Lottery Sales.

Figure 13 illustrates the quantitative impact of varying public holidays on lottery sales derived from the model components. It shows that National Day and Labor Day have the most substantial effects on lottery sales, with absolute impact values of 0.022793 and 0.019517, respectively. These holidays were followed by Spring Festival and New Year’s Day, which displayed smaller, though still noticeable, average deviations.

To validate whether these holiday-induced variations are statistically significant, a series of hypothesis tests were conducted for each holiday. Table 6 summarizes the modeled effect size, the absolute average impact, and the corresponding *p*-value obtained from the hypothesis test. Table 6 reveals heterogeneous impacts across different holidays. National Day and New Year’s Day showed statistically significant negative coefficients, indicating a marked decline in sales deviation relative to the baseline. In contrast, Labor Day exhibited a significant positive impact. These statistically significant deviations confirm that calendar events are strong drivers of sales volatility, inducing consistent consumer behavior changes that vary in direction depending on the specific holiday context.

Conversely, some culturally important holidays, such as the Spring Festival, Dragon Boat Festival, and Mid-Autumn Festival, were found to have statistically insignificant effects. For example, the Spring Festival, while traditionally seen as a major national event, had a *p*-value of 0.381838, suggesting that its effect on sales was not statistically distinguishable from random fluctuations. This might have been due to business closures or a shift in consumer spending away from lottery-related activities during the holiday. Similarly, the Mid-Autumn Festival and Dragon Boat Festival yielded *p*-values of 0.113051 and 0.395270, respectively, indicating that their influence on sales was weak or inconsistent.

These findings highlight the need to treat public holidays as heterogeneous in their economic impact. While some holidays produce clear and statistically robust effects on consumer behavior, others show minimal or inconsistent influence. For forecasting models, particularly those involving long-term sales predictions, integrating holidays with demonstrated statistical significance—such as National Day and Labor Day—can meaningfully enhance model accuracy and reliability. Furthermore, from a managerial or strategic perspective, these results may inform more targeted promotional campaigns or resource allocation during high-impact holiday seasons.

#### 4.2.2. Seasonal Effects

To quantitatively assess the influence of seasonal cycle length on forecasting performance, we compared model accuracy under annual (m = 12), biennial (m = 24), triennial (m = 36), and quadrennial (m = 48) seasonal settings. The quadrennial configuration was motivated by an event-induced long-cycle seasonality hypothesis, where lottery sales exhibit structural surges approximately every four years, coinciding with the FIFA World Cup.

Table 7 presents the in-sample fit (R^2^) and out-of-sample forecast metrics (MAPE, SMAPE, and 90th percentile error) for each seasonal cycle length. The results indicate that the 48-month cycle demonstrates a dominant advantage across almost all key indicators. Specifically, the 48-month cycle achieved the highest correlation and the lowest average errors, recording a MAPE of 6.51%. This stands in sharp contrast to the 12-, 24-, and 36-month cycles, all of which yielded MAPEs exceeding 11%, suggesting that shorter cycles fail to adequately capture the long-term structural fluctuations driven by mega-events such as the World Cup.

Regarding the capability to capture extreme values (90th percentile error), although the 24-month cycle exhibited a slightly lower error, its overall predictive accuracy was poor, implying excessive smoothing of sales volatility. In contrast, the 48-month cycle not only significantly improved overall accuracy but also maintained the extreme value error at a highly competitive level of 7.50%. This dual advantage of high precision and robustness confirms the existence of a distinct four-year periodicity in the Chinese lottery market. Consequently, setting the seasonal period to 48 months proves to be the optimal choice for modeling these market dynamics.

Based on the performance evaluations and seasonal effect analyses, the subsequent section draws conclusions from the findings and outlines potential directions for future research, emphasizing the practical implications of the proposed hybrid forecasting approach.

## 5. Conclusions and Future Work

This study conducted a comparative analysis of three time series forecasting models—SARIMA, Prophet, and the proposed DDC-Hybrid—aiming to enhance the accuracy of lottery sales predictions for decision support in lottery administration. The results show that the DDC-Hybrid consistently yielded lower error metrics than both individual models across all tested scenarios. More importantly, this study validates the effectiveness of the proposed structured diagnostic workflow. Our findings confirm that statistical residual diagnosis is a critical prerequisite for hybrid modeling, as it scientifically justifies the integration of SARIMA to capture the short-term dependencies left unexplained by Prophet. This explicit justification distinguishes our approach from arbitrary model ensembles. The sales projections for 2026 to 2028 indicated a steady upward trend, confirming the model’s reliability in capturing underlying market dynamics.

Despite its solid performance, the DDC-Hybrid has certain limitations. First, regarding generalizability, this study relies exclusively on aggregated national-level data. While sufficient for macro-strategic planning, empirical evidence regarding the method’s applicability across heterogeneous regions, specific product types, or finer temporal resolutions remains absent. Second, the model currently lacks exogenous variables, limiting its responsiveness to sudden external shocks. Finally, although the integration of GARCH mitigates volatility risks, the linear SARIMA component may still struggle to fully capture complex nonlinear feature interactions compared to non-parametric methods.

Future research will prioritize two key directions to address these gaps. First, to verify generalizability, we plan to apply this diagnostic pipeline to disaggregated time series—specifically provincial-level data and distinct categories—to test its robustness across diverse market segments. Second, we aim to enhance model comparison and non-linearity modeling. Future iterations will benchmark advanced deep learning architectures, including LSTM, GRU, Multilayer Perceptron (MLP), and Temporal Convolutional Networks (TCN), as potential alternatives to the current Prophet or SARIMA components. These models will be rigorously evaluated to determine if their capacity for learning high-dimensional nonlinear dependencies offers a statistically significant improvement over the proposed diagnostic hybrid framework.

## Figures and Tables

**Table 1:** Summary of Key Forecasting Models and the Proposed DDC-Hybrid Framework.

Model	Methodology	Application Context	Performance	Limitations
SARIMA	Trend + seasonality via ARIMA	Electricity, stock, retail	Moderate	Requires stationary data, linear assumptions
Prophet	Additive trend + seasonality + holidays	Website traffic, sales, finance	High for long-term	Less effective for irregular short-term data
LSTM	Deep learning with memory capability	Cryptocurrency, weather, finance	High	Data-hungry, computationally intensive
Hybrid SARIMA-Prophet	Combine Prophet trend with SARIMA residuals	Epidemics, e-commerce	Often high accuracy reported	Often lacks diagnostic justification for mixing
NeuralProphet	Prophet + neural autoregression	Large-scale business, forecasting tasks	Competitive	Still experimental, less validated
DDC-Hybrid	Diagnostic pipeline	Long-horizon, multi-scale seasonality	Improved accuracy	Multi-stage complexity

**Table 2:** Top 10 SARIMA Models by AICc Value.

AR	I	MA	SAR	SI	SMA	Seasonality	AICc Value
0	1	1	2	0	0	48	−187.478
0	1	3	2	0	0	48	−186.776
1	1	2	2	0	0	48	−186.493
0	1	2	2	0	0	48	−186.466
0	1	1	2	0	1	48	−186.361
0	1	1	1	0	2	48	−186.358
2	1	1	2	0	0	48	−186.156
1	1	1	2	0	0	48	−186.107
0	1	2	2	0	1	48	−184.514
0	1	3	1	0	1	48	−184.194

**Table 3:** Prophet Holiday Definition Table.

Holiday	Date	Lower Window (Days)	Upper Window (Days)
Spring Festival	The first day of the first lunar month	−7	7
National Day	October 1	−5	5
Mid-Autumn Festival	The 15th day of the eighth lunar month	−3	3
Labor Day	May 1	−3	3
Dragon Boat Festival	The fifth day of the fifth lunar month	−2	2
New Year’s Day	January 1	0	1

**Table 4:** Prophet hyperparameter tuning results.

Changepoint_Prior_Scale	Fourier_Order	MAPE (%)
0.03	5	7.35
0.03	10	9.11
0.03	15	6.98
0.05	5	7.81
0.05	10	8.07
0.05	15	6.81
0.08	5	7.72
0.08	10	16.71
0.08	15	15.96

**Table 5:** Model Evaluation Metrics. (All metrics are computed on the original scale of sales volume).

Model	R^2^ (In-Sample)	MAPE (%) (Out-of-Sample)	SMAPE (%) (Out-of-Sample)	MSE (Billion CNY)^2^ (Out-of-Sample)
SARIMA	0.8554	7.22	6.87	18.67
Prophet	0.9315	6.81	6.56	19.59
DDC-Hybrid	0.9515	6.51	6.23	18.23

**Table 6:** Effects of Holidays, Absolute Impact, and Statistical Significance.

Holidays	Effect	Absolute Effect	*p*-Value
National Day	−0.022793	0.022793	<0.001
Labor Day	0.019517	0.019517	<0.001
Spring Festival	−0.002285	0.006300	0.381838
New Year’s Day	−0.006020	0.006020	<0.001
Dragon Boat Festival	−0.001316	0.002319	0.395270
Mid-Autumn Festival	0.000966	0.001193	0.113051

**Table 7:** Performance Comparison Across Different Seasonal Cycle Lengths.

Seasonality Period (Months)	R^2^	MAPE (%)	SMAPE (%)	90th Percentile Error (%)
12	0.9475	11.58	11.78	22.31
24	0.9492	13.77	12.63	6.20
36	0.9420	13.48	13.02	11.84
48	0.9515	6.51	6.23	7.50

## Data Availability

The data and code used to support the findings of this study are available in the Supplementary Materials.

## Supplementary Materials

The following supporting information can be downloaded at: https://www.mdpi.com/article/10.3390/e28030286/s1, supplementary dataset and the Python code used for data preprocessing, model training, and figure generation.

## Abbreviations

The following abbreviations are used in this manuscript:

SARIMA	Seasonal Autoregressive Integrated Moving Average
DDC-Hybrid	Diagnostic Decomposition–Correction Hybrid
ACF	Autocorrelation Function
PACF	Partial Autocorrelation Function
ADF	Augmented Dickey–Fuller (test)
AIC	Akaike Information Criterion
R^2^	R-squared (Coefficient of Determination)
MAPE	Mean Absolute Percentage Error
SMAPE	Symmetric Mean Absolute Percentage Error
CNY	Chinese Yuan
